# 1-Iodo­triptycene

**DOI:** 10.1107/S160053681103279X

**Published:** 2011-08-17

**Authors:** Richard Betz, Cedric McCleland, André Scheffer

**Affiliations:** a Nelson Mandela Metropolitan University, Summerstrand Campus, Department of Chemistry, University Way, Summerstrand, PO Box 77000, Port Elizabeth, 6031, South Africa

## Abstract

The title compound, C_20_H_13_I, is a halogenated derivative of triptycene. The mol­ecule shows crystallographic as well as non-crystallographic *C*_3_ symmetry. The asymmetric unit comprises one third of the mol­ecule. Dispersive I⋯I contacts [I⋯I = 3.6389 (3) Å] connect the mol­ecules into dimers. The shortest centroid–centroid distance between two π-systems is 3.8403 (12) Å.

## Related literature

For the crystal structures of 1-bromo­triptycene, 9,10-di­bromo­triptycene and 10-bromo-9-triptycyl iodo­formate, see: Palmer & Templeton (1968 ▶), Abergel & Dinca (2004 ▶) and de Wet *et al.* (1978 ▶), respectively. For the preparation, see: Bartel *et al.* (1971 ▶).
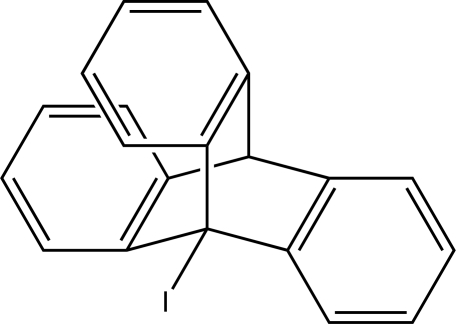


## Experimental

### 

#### Crystal data

C_20_H_13_I
*M* *_r_* = 380.20
Hexagonal, 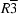

*a* = 11.8820 (4) Å
*c* = 17.6800 (5) Å
*V* = 2161.68 (12) Å^3^
*Z* = 6
Mo *K*α radiation
μ = 2.21 mm^−1^
*T* = 200 K
0.56 × 0.51 × 0.25 mm

#### Data collection

Bruker APEXII CCD diffractometer
Absorption correction: multi-scan (*SADABS*; Bruker, 2008 ▶) *T*_min_ = 0.568, *T*_max_ = 0.746
4033 measured reflections
1184 independent reflections
1156 reflections with *I* > 2σ(*I*)
*R*_int_ = 0.011

#### Refinement

*R*[*F* ^2^ > 2σ(*F* ^2^)] = 0.021
*wR*(*F* ^2^) = 0.059
*S* = 1.15
1184 reflections
64 parameters
H-atom parameters constrained
Δρ_max_ = 1.52 e Å^−3^
Δρ_min_ = −0.51 e Å^−3^

### 

Data collection: *APEX2* (Bruker, 2010 ▶); cell refinement: *SAINT* (Bruker, 2010 ▶); data reduction: *SAINT*; program(s) used to solve structure: *SIR97* (Altomare *et al.*, 1999 ▶); program(s) used to refine structure: *SHELXL97* (Sheldrick, 2008 ▶); molecular graphics: *ORTEP-3* (Farrugia, 1997 ▶); software used to prepare material for publication: *SHELXL97* and *PLATON* (Spek, 2009 ▶).

## Supplementary Material

Crystal structure: contains datablock(s) I, global. DOI: 10.1107/S160053681103279X/nk2107sup1.cif

Supplementary material file. DOI: 10.1107/S160053681103279X/nk2107Isup2.cdx

Structure factors: contains datablock(s) I. DOI: 10.1107/S160053681103279X/nk2107Isup3.hkl

Additional supplementary materials:  crystallographic information; 3D view; checkCIF report

## Figures and Tables

**Fig. 1.**
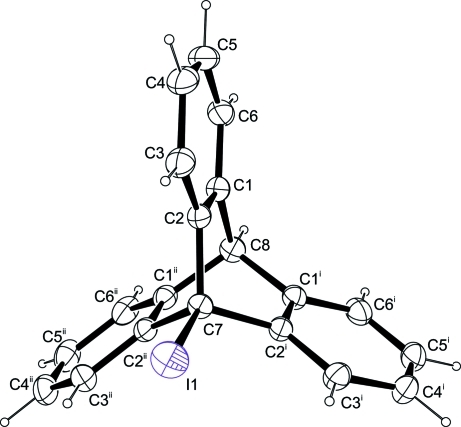
The molecular structure of the title compound, anisotropic displacement ellipsoids are drawn at 50% probability level. Symmetry operators: i -y, x-y, z; ii -x + y, -x, z.

**Fig. 2.**
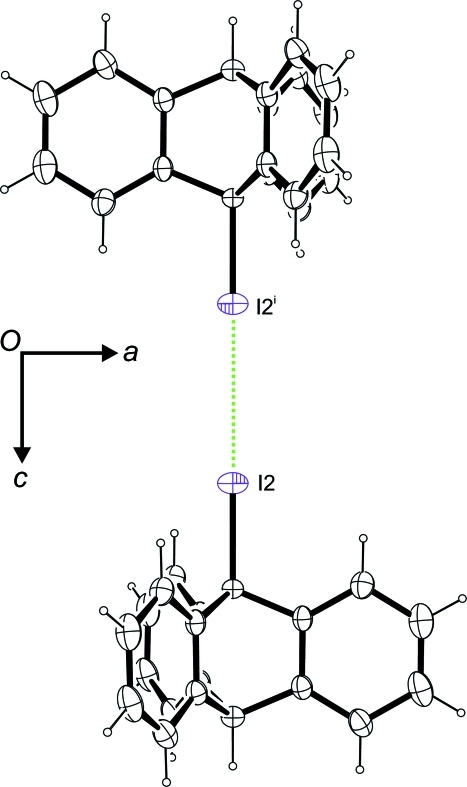
Intermolecular I···I contact, viewed along [0 - 1 0]. Symmetry operator: i -x, -y, -z.

## supplementary crystallographic information

### Comment

The chemistry of molecules featuring double and triple bonds involving elements from the third row of the periodic system of the elements (or below) is affected by the marked tendency of oligo- and polymerization. The introduction of sterically demanding, "bulky" protective groups in proximity to such bonding systems allowed the isolation and characterization of respective compounds on grounds of steric shielding and, as a consequence, markedly decreased rate of polymerization. It seemed of interest for us to study whether the presence of such aforementioned bonding systems has an influence on the metrical parameters of the applied protection groups as well. Therefore, we determined the crystal structure of the title compound. So far, the molecular and crystal stuctures of 1-bromotriptycene (Palmer & Templeton, 1968), 9,10-dibromotriptycene (Abergel & Dinca, 2004) as well as 10-bromo-9-triptycyl iodoformate (de Wet *et al.*, 1978) are the only examples of structurally characterized triptycene compounds bearing a halogenido substituent on the bridgehead carbon atom present in the literature.

Halogenation took place on one of the bridgehead carbon atoms of the triptycene molecule (Figure 1). The least-squares planes defined by the atoms of the three aromatic moieties enclose angles of 60.03 (4) ° and 60.03 (7), respectively.

In the molecules, dispersive I···I contacts whose range falls by more than 0.3 Å below the sum of van der Waals radii can be observed (Figure 2). These connect two molecules to dimeric units whose I···I vector is pointing along the crystallographic *c* axis. The aromatic moieties of one molecule in such a dimer adopt a staggered conformation towards the aromatic moieties in the other molecule when projected along the I···I axis. The closest intercentroid distance between two π-systems was measured at 3.8403 (12) Å.

### Experimental

The compound was formed through the thermolysis of 9-triptycyl iodoformate according to a published procedure (Bartel *et al.*, 1971).

### Refinement

Carbon-bound H atoms were placed in calculated positions (C—H 0.95 Å for aromatic carbon atoms, C—H 1.00 Å for the bridgehead carbon atom) and were included in the refinement in the riding model approximation, with *U*(H) set to 1.2*U*_eq_(C).

### Crystal data

**Table:**

C_20_H_13_I	*D*_x_ = 1.752 Mg m^−^^3^
*M**_r_* = 380.20	Mo *K*α radiation, λ = 0.71069 Å
Hexagonal, *R*3	Cell parameters from 3561 reflections
Hall symbol: -R 3	θ = 4.1–28.3°
*a* = 11.8820 (4) Å	µ = 2.21 mm^−^^1^
*c* = 17.6800 (5) Å	*T* = 200 K
*V* = 2161.68 (12) Å^3^	Block, colourless
*Z* = 6	0.56 × 0.51 × 0.25 mm
*F*(000) = 1116	

### Data collection

**Table:**

Bruker APEXII CCD diffractometer	1184 independent reflections
Radiation source: fine-focus sealed tube	1156 reflections with *I* > 2σ(*I*)
graphite	*R*_int_ = 0.011
φ and ω scans	θ_max_ = 28.3°, θ_min_ = 3.5°
Absorption correction: multi-scan (*SADABS*; Bruker, 2008)	*h* = −15→10
*T*_min_ = 0.568, *T*_max_ = 0.746	*k* = −13→15
4033 measured reflections	*l* = −20→23

### Refinement

**Table:**

Refinement on *F*^2^	Primary atom site location: structure-invariant direct methods
Least-squares matrix: full	Secondary atom site location: difference Fourier map
*R*[*F*^2^ > 2σ(*F*^2^)] = 0.021	Hydrogen site location: inferred from neighbouring sites
*wR*(*F*^2^) = 0.059	H-atom parameters constrained
*S* = 1.15	*w* = 1/[σ^2^(*F*_o_^2^) + (0.0366*P*)^2^ + 2.801*P*] where *P* = (*F*_o_^2^ + 2*F*_c_^2^)/3
1184 reflections	(Δ/σ)_max_ < 0.001
64 parameters	Δρ_max_ = 1.52 e Å^−^^3^
0 restraints	Δρ_min_ = −0.51 e Å^−^^3^

### Fractional atomic coordinates and isotropic or equivalent isotropic displacement parameters (Å^2^)

**Table:**

	*x*	*y*	*z*	*U*_iso_*/*U*_eq_	
I1	0.0000	0.0000	0.102907 (11)	0.03354 (10)	
C1	0.11904 (15)	0.11668 (15)	0.33674 (10)	0.0219 (3)	
C2	0.12090 (15)	0.11714 (15)	0.25794 (10)	0.0207 (3)	
C3	0.22522 (17)	0.21626 (17)	0.21950 (11)	0.0264 (3)	
H3	0.2274	0.2169	0.1658	0.032*	
C4	0.32674 (18)	0.31494 (17)	0.26061 (13)	0.0330 (4)	
H4	0.3988	0.3826	0.2346	0.040*	
C5	0.32397 (18)	0.31565 (18)	0.33846 (14)	0.0337 (4)	
H5	0.3933	0.3842	0.3657	0.040*	
C6	0.21961 (18)	0.21594 (17)	0.37755 (12)	0.0284 (4)	
H6	0.2174	0.2160	0.4313	0.034*	
C7	0.0000	0.0000	0.22412 (15)	0.0188 (5)	
C8	0.0000	0.0000	0.37075 (17)	0.0224 (5)	
H8	0.0000	0.0000	0.4273	0.027*	

### Atomic displacement parameters (Å^2^)

**Table:**

	*U*^11^	*U*^22^	*U*^33^	*U*^12^	*U*^13^	*U*^23^
I1	0.04045 (12)	0.04045 (12)	0.01971 (13)	0.02022 (6)	0.000	0.000
C1	0.0206 (7)	0.0206 (7)	0.0262 (8)	0.0115 (6)	−0.0022 (6)	−0.0016 (6)
C2	0.0177 (7)	0.0176 (7)	0.0276 (8)	0.0094 (6)	−0.0006 (6)	−0.0004 (6)
C3	0.0230 (7)	0.0231 (7)	0.0318 (9)	0.0107 (6)	0.0039 (6)	0.0044 (6)
C4	0.0212 (8)	0.0203 (8)	0.0530 (12)	0.0071 (6)	0.0005 (7)	0.0042 (8)
C5	0.0238 (8)	0.0206 (7)	0.0538 (12)	0.0089 (7)	−0.0117 (8)	−0.0056 (8)
C6	0.0280 (8)	0.0254 (8)	0.0349 (9)	0.0157 (7)	−0.0098 (7)	−0.0072 (7)
C7	0.0202 (7)	0.0202 (7)	0.0162 (12)	0.0101 (4)	0.000	0.000
C8	0.0242 (8)	0.0242 (8)	0.0189 (13)	0.0121 (4)	0.000	0.000

### Geometric parameters (Å, °)

**Table:**

I1—C7	2.143 (3)	C4—H4	0.9500
C1—C6	1.389 (2)	C5—C6	1.396 (3)
C1—C2	1.393 (2)	C5—H5	0.9500
C1—C8	1.524 (2)	C6—H6	0.9500
C2—C3	1.388 (2)	C7—C2^i^	1.5359 (19)
C2—C7	1.5359 (19)	C7—C2^ii^	1.5359 (19)
C3—C4	1.394 (3)	C8—C1^i^	1.524 (2)
C3—H3	0.9500	C8—C1^ii^	1.524 (2)
C4—C5	1.377 (4)	C8—H8	1.0000
			
C6—C1—C2	120.71 (16)	C1—C6—C5	119.03 (19)
C6—C1—C8	125.48 (18)	C1—C6—H6	120.5
C2—C1—C8	113.81 (16)	C5—C6—H6	120.5
C3—C2—C1	119.92 (16)	C2—C7—C2^i^	105.82 (13)
C3—C2—C7	127.75 (17)	C2—C7—C2^ii^	105.82 (13)
C1—C2—C7	112.33 (16)	C2^i^—C7—C2^ii^	105.82 (13)
C2—C3—C4	119.24 (18)	C2—C7—I1	112.92 (11)
C2—C3—H3	120.4	C2^i^—C7—I1	112.92 (11)
C4—C3—H3	120.4	C2^ii^—C7—I1	112.92 (11)
C5—C4—C3	120.87 (17)	C1^i^—C8—C1^ii^	105.46 (14)
C5—C4—H4	119.6	C1^i^—C8—C1	105.46 (14)
C3—C4—H4	119.6	C1^ii^—C8—C1	105.46 (14)
C4—C5—C6	120.22 (17)	C1^i^—C8—H8	113.2
C4—C5—H5	119.9	C1^ii^—C8—H8	113.2
C6—C5—H5	119.9	C1—C8—H8	113.2
			
C6—C1—C2—C3	1.2 (2)	C3—C2—C7—C2^i^	122.9 (2)
C8—C1—C2—C3	−178.53 (13)	C1—C2—C7—C2^i^	−56.63 (15)
C6—C1—C2—C7	−179.19 (13)	C3—C2—C7—C2^ii^	−125.1 (2)
C8—C1—C2—C7	1.07 (16)	C1—C2—C7—C2^ii^	55.38 (16)
C1—C2—C3—C4	−0.4 (2)	C3—C2—C7—I1	−1.06 (17)
C7—C2—C3—C4	−179.98 (14)	C1—C2—C7—I1	179.37 (9)
C2—C3—C4—C5	−0.6 (3)	C6—C1—C8—C1^i^	−124.7 (2)
C3—C4—C5—C6	0.9 (3)	C2—C1—C8—C1^i^	55.02 (16)
C2—C1—C6—C5	−0.9 (2)	C6—C1—C8—C1^ii^	124.0 (2)
C8—C1—C6—C5	178.79 (14)	C2—C1—C8—C1^ii^	−56.30 (16)
C4—C5—C6—C1	−0.1 (3)		

Symmetry codes: (i) −*x*+*y*, −*x*, *z*; (ii) −*y*, *x*−*y*, *z*.
